# (2,2′-Bipyrid­yl)bis­[*N*-(2-hydroxy­ethyl)-*N*-*n*-propyl­dithio­carbamato-κ^2^ *S*,*S*′]cadmium(II) acetonitrile solvate

**DOI:** 10.1107/S1600536809049666

**Published:** 2009-11-25

**Authors:** Juyoung C. Song, Edward R. T. Tiekink

**Affiliations:** a Department of Chemistry, The University of Texas at San Antonio, One UTSA Circle, San Antonio, Texas 78249-0698, USA; b Department of Chemistry, University of Malaya, 50603 Kuala Lumpur, Malaysia

## Abstract

The title complex, [Cd(C_6_H_12_NOS_2_)_2_(C_10_H_8_N_2_)]·CH_3_CN, features a distorted octa­hedral N_2_S_4_ geometry for the Cd^II^ centre defined by a pair of asymmetrically chelating dithio­carbamate ligands as well as a 2,2′-bipyridine ligand. Supra­molecular chains along [001] are formed in the crystal structure, mediated by O—H⋯S hydrogen bonds; the acetonitrile solvent mol­ecules are associated with the chains *via* O—H⋯N hydrogen bonds.

## Related literature

For background to supra­molecular polymers of zinc-triad 1,1-dithiol­ates, see: Tiekink (2003 ▶); Lai *et al.* (2002 ▶); Chen *et al.* (2006 ▶); Benson *et al.* (2007 ▶). For the synthesis, see: Lai & Tiekink (2004 ▶).
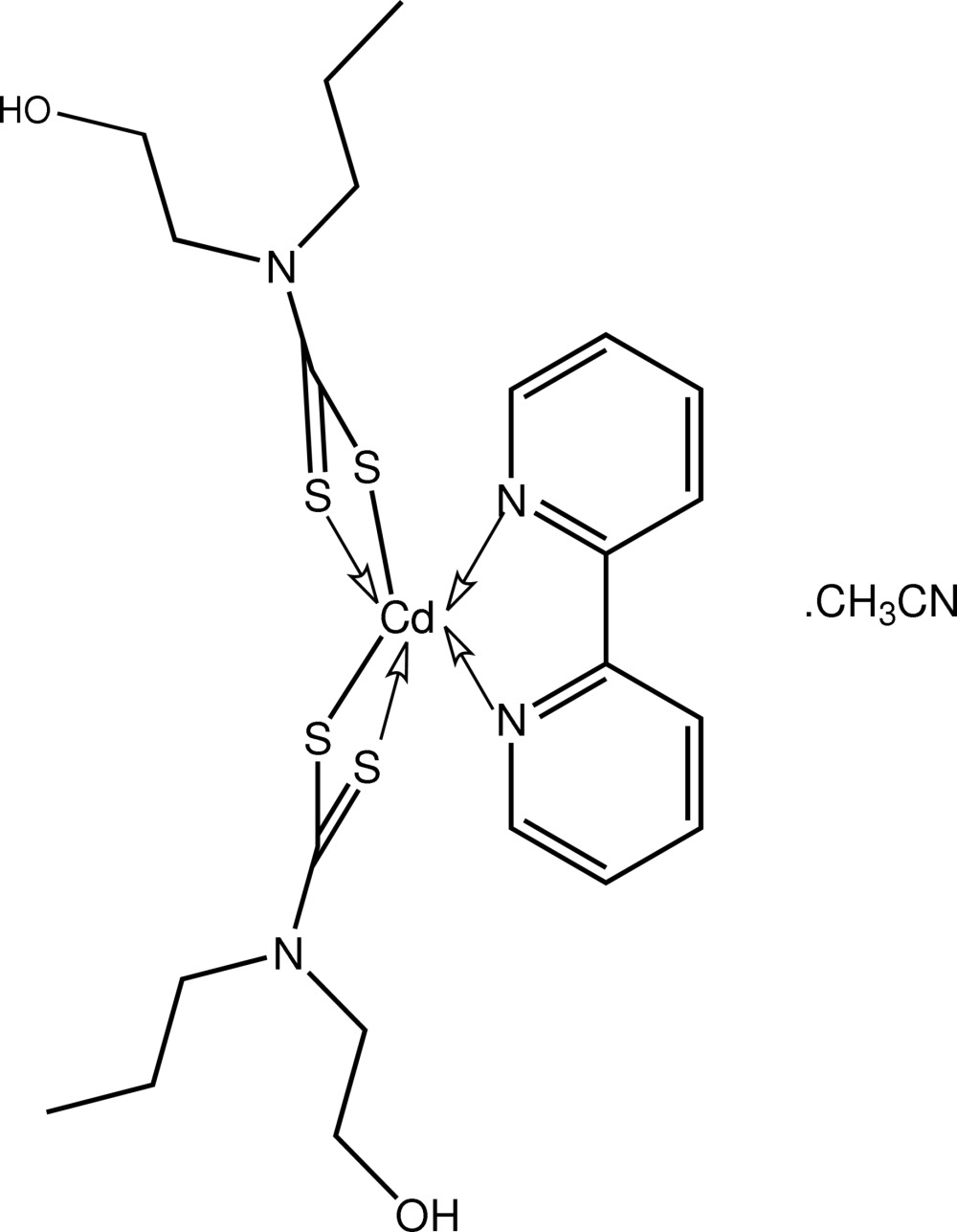


## Experimental

### 

#### Crystal data

[Cd(C_6_H_12_NOS_2_)_2_(C_10_H_8_N_2_)]·C_2_H_3_N
*M* *_r_* = 666.21
Monoclinic, 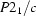

*a* = 7.3277 (7) Å
*b* = 23.822 (2) Å
*c* = 17.1159 (18) Å
β = 99.786 (1)°
*V* = 2944.2 (5) Å^3^
*Z* = 4
Mo *K*α radiation
μ = 1.06 mm^−1^
*T* = 98 K
0.36 × 0.22 × 0.11 mm

#### Data collection

Rigaku AFC12K/SATURN724 diffractometer
Absorption correction: multi-scan (*ABSCOR*; Higashi, 1995 ▶) *T*_min_ = 0.828, *T*_max_ = 1
18201 measured reflections
6043 independent reflections
5711 reflections with *I* > 2σ(*I*)
*R*_int_ = 0.027

#### Refinement

*R*[*F* ^2^ > 2σ(*F* ^2^)] = 0.030
*wR*(*F* ^2^) = 0.080
*S* = 1.09
6043 reflections
331 parameters
2 restraints
H-atom parameters constrained
Δρ_max_ = 0.70 e Å^−3^
Δρ_min_ = −0.69 e Å^−3^

### 

Data collection: *CrystalClear* (Rigaku/MSC, 2005 ▶); cell refinement: *CrystalClear*; data reduction: *CrystalClear*; program(s) used to solve structure: *PATTY* in *DIRDIF92* (Beurskens *et al.*, 1992 ▶); program(s) used to refine structure: *SHELXL97* (Sheldrick, 2008 ▶); molecular graphics: *ORTEPII* (Johnson, 1976 ▶) and *DIAMOND* (Brandenburg, 2006 ▶); software used to prepare material for publication: *publCIF* (Westrip, 2009 ▶).

## Supplementary Material

Crystal structure: contains datablocks global, I. DOI: 10.1107/S1600536809049666/hb5240sup1.cif

Structure factors: contains datablocks I. DOI: 10.1107/S1600536809049666/hb5240Isup2.hkl

Additional supplementary materials:  crystallographic information; 3D view; checkCIF report

## Figures and Tables

**Table 1:** Selected bond lengths (Å)

Cd—N3	2.361 (2)
Cd—N4	2.406 (2)
Cd—S1	2.5872 (7)
Cd—S3	2.6539 (7)
Cd—S4	2.6704 (7)
Cd—S2	2.7816 (7)

**Table 2:** Hydrogen-bond geometry (Å, °)

*D*—H⋯*A*	*D*—H	H⋯*A*	*D*⋯*A*	*D*—H⋯*A*
O1—H1o⋯N5	0.84	2.06	2.898 (3)	174
O2—H2o⋯S2^i^	0.84	2.55	3.388 (2)	175

Symmetry code: (i) 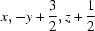.

**Fig. 1.**
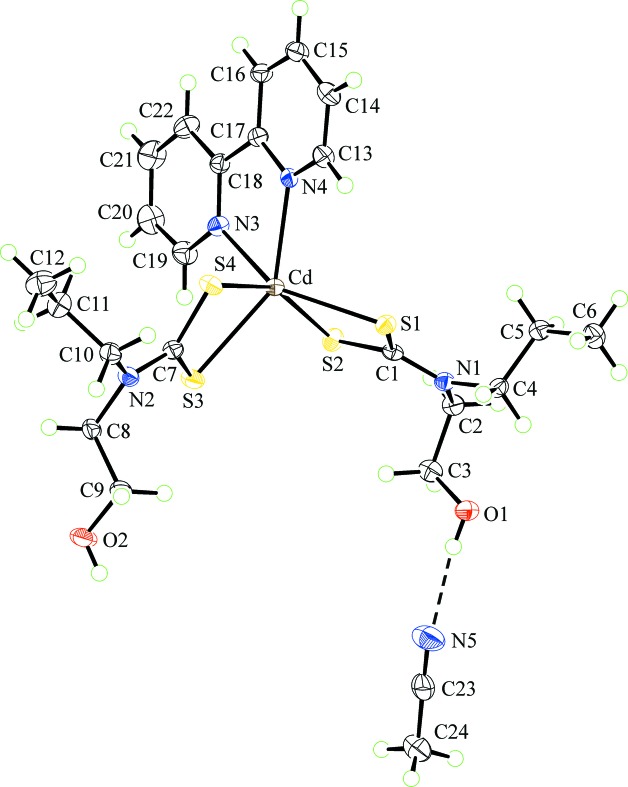
Molecular structure of (I) showing displacement ellipsoids at the 50% probability level. The O–H···N hydrogen bond is shown as a dashed line.

**Fig. 2.**
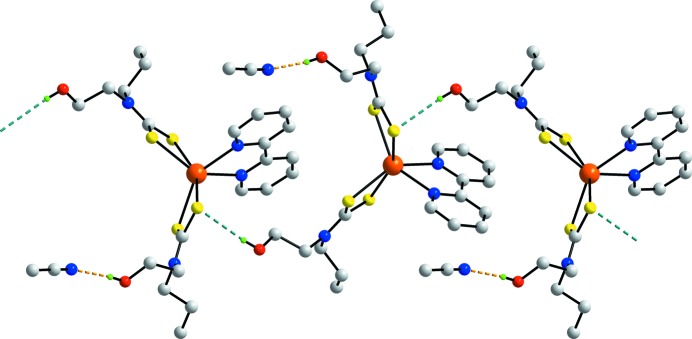
Supramolecular chain in (I) mediated by O–H···S (green dashed lines) hydrogen bonds. The solvent acetonitrile molecules are connected by O–H···N hydrogen bonds (orange dashed lines). Hydrogen atoms not involved in the hydrogen bonding are omitted for reasons of clarity. Colour code: Cd, orange; S, yellow; O, red; N, blue; C, grey; and H, green.

## supplementary crystallographic information

### Comment

Crystal engineering studies on the zinc-triad 1,1-dithiolates have generated 1-D, 2-D and 3-D architectures (Lai *et al.*, 2002; Tiekink, 2003; Chen *et al.*, 2006), in particular with dithiocarbamate ligands functionalized with hydrogen-bonding capacity (Benson *et al.*, 2007). As a continuation of studies in this field, the structure of the title compound, (I), was investigated.

The molecular structure of (I) features a hexa-coordinated Cd^II^ centre defined by two asymmetrically chelating dithiocarbamate ligands (Cd–S1, S2 = 2.5872 (7) and 2.7816 (7) Å, and Cd–S3, S4 = 2.6539 (7) and 2.6704 (7) Å) and a chelating 2,2'-bipyridyl ligand (Cd–N3, N4 = 2.361 (2), 2.406 (2) Å). The resulting N_2_S_4_ donor set defines a distorted octahedral geometry.

The crystal packing is dominated by O–H···O and O–H···N hydrogen bonds, Table 1. The latter involve the O1-hydroxyl group and the nitrile-N5 atom of the solvent acetonitrile molecule. The O2-hydroxyl group forms hydrogen bonds with the dithiocarbamate-S2 atom to generate a supramolecular chain along [0 0 1], Table 1 and Fig. 2.

### Experimental

Compound (I) was prepared following the standard literature procedure from the reaction of Cd[S_2_CN(CH_2_CH_2_OH)(n-Pr)]_2_ and 2,2'-bipyridyl (Lai & Tiekink, 2004). Colourless crystals were obtained from the slow evaporation of an acetonitrile solution of (I). IR (KBr, cm^-1^): 1471 (*m*) ν(C=N); 1183 (*s*) ν(C—S).

### Refinement

C-bound H-atoms were placed in calculated positions (C–H 0.95–0.99 Å) and were included in the refinement in the riding model approximation with *U*_iso_(H) set to 1.2*U*_eq_(C). A rotating group model was used for the methyl groups. The O-bound H-atoms were located in a difference Fourier map and each refined with an O–H restraint of 0.840±0.001 Å, and with *U*_iso_(H) = 1.5*U*_eq_(carrier atom).

### Crystal data

**Table:**

[Cd(C_6_H_12_NOS_2_)_2_(C_10_H_8_N_2_)]·C_2_H_3_N	*F*(000) = 1368
*M**_r_* = 666.21	*D*_x_ = 1.503 Mg m^−^^3^
Monoclinic, *P*2_1_/*c*	Mo *K*α radiation, λ = 0.71069 Å
Hall symbol: -P 2ybc	Cell parameters from 18141 reflections
*a* = 7.3277 (7) Å	θ = 2.1–40.6°
*b* = 23.822 (2) Å	µ = 1.06 mm^−^^1^
*c* = 17.1159 (18) Å	*T* = 98 K
β = 99.786 (1)°	Block, colourless
*V* = 2944.2 (5) Å^3^	0.36 × 0.22 × 0.11 mm
*Z* = 4	

### Data collection

**Table:**

Rigaku AFC12K/SATURN724 diffractometer	6043 independent reflections
Radiation source: fine-focus sealed tube	5711 reflections with *I* > 2σ(*I*)
graphite	*R*_int_ = 0.027
ω scans	θ_max_ = 26.5°, θ_min_ = 2.1°
Absorption correction: multi-scan (*ABSCOR*; Higashi, 1995)	*h* = −9→9
*T*_min_ = 0.828, *T*_max_ = 1	*k* = −25→29
18201 measured reflections	*l* = −21→21

### Refinement

**Table:**

Refinement on *F*^2^	Primary atom site location: structure-invariant direct methods
Least-squares matrix: full	Secondary atom site location: difference Fourier map
*R*[*F*^2^ > 2σ(*F*^2^)] = 0.030	Hydrogen site location: inferred from neighbouring sites
*wR*(*F*^2^) = 0.080	H-atom parameters constrained
*S* = 1.09	*w* = 1/[σ^2^(*F*_o_^2^) + (0.0396*P*)^2^ + 3.165*P*] where *P* = (*F*_o_^2^ + 2*F*_c_^2^)/3
6043 reflections	(Δ/σ)_max_ = 0.004
331 parameters	Δρ_max_ = 0.70 e Å^−^^3^
2 restraints	Δρ_min_ = −0.69 e Å^−^^3^

### Special details

**Table:**

Geometry. All e.s.d.'s (except the e.s.d. in the dihedral angle between two l.s. planes) are estimated using the full covariance matrix. The cell e.s.d.'s are taken into account individually in the estimation of e.s.d.'s in distances, angles and torsion angles; correlations between e.s.d.'s in cell parameters are only used when they are defined by crystal symmetry. An approximate (isotropic) treatment of cell e.s.d.'s is used for estimating e.s.d.'s involving l.s. planes.
Refinement. Refinement of *F*^2^ against ALL reflections. The weighted *R*-factor *wR* and goodness of fit *S* are based on *F*^2^, conventional *R*-factors *R* are based on *F*, with *F* set to zero for negative *F*^2^. The threshold expression of *F*^2^ > σ(*F*^2^) is used only for calculating *R*-factors(gt) *etc*. and is not relevant to the choice of reflections for refinement. *R*-factors based on *F*^2^ are statistically about twice as large as those based on *F*, and *R*- factors based on ALL data will be even larger.

### Fractional atomic coordinates and isotropic or equivalent isotropic displacement parameters (Å^2^)

**Table:**

	*x*	*y*	*z*	*U*_iso_*/*U*_eq_	
Cd	0.22264 (2)	0.757722 (7)	0.046853 (9)	0.01758 (7)	
S1	0.17585 (8)	0.85953 (3)	0.09227 (3)	0.01984 (13)	
S2	0.54757 (9)	0.82033 (3)	0.06550 (4)	0.02253 (13)	
S3	0.35255 (9)	0.69177 (3)	0.16861 (4)	0.02338 (14)	
S4	−0.04308 (9)	0.69484 (3)	0.09167 (3)	0.02234 (13)	
O1	0.7384 (3)	0.95516 (8)	0.26074 (11)	0.0274 (4)	
H1O	0.8084	0.9465	0.3031	0.041*	
O2	0.4126 (3)	0.60342 (9)	0.39861 (11)	0.0356 (5)	
H2O	0.4471	0.6205	0.4415	0.053*	
N1	0.4822 (3)	0.92222 (9)	0.11936 (11)	0.0184 (4)	
N2	0.0887 (3)	0.62810 (9)	0.21271 (12)	0.0215 (4)	
N3	0.3447 (3)	0.70592 (9)	−0.04998 (12)	0.0205 (4)	
N4	0.0208 (3)	0.76057 (8)	−0.07961 (12)	0.0177 (4)	
N5	0.9711 (4)	0.93255 (13)	0.41198 (16)	0.0471 (8)	
C1	0.4102 (3)	0.87219 (10)	0.09512 (13)	0.0183 (5)	
C2	0.6792 (3)	0.93562 (11)	0.12198 (14)	0.0212 (5)	
H2A	0.6918	0.9762	0.1116	0.025*	
H2B	0.7258	0.9146	0.0795	0.025*	
C3	0.7967 (4)	0.92111 (12)	0.20105 (15)	0.0250 (5)	
H3A	0.7818	0.8809	0.2132	0.030*	
H3B	0.9290	0.9282	0.1991	0.030*	
C4	0.3645 (3)	0.96886 (10)	0.13808 (13)	0.0195 (5)	
H4A	0.4397	0.9948	0.1758	0.023*	
H4B	0.2640	0.9538	0.1640	0.023*	
C5	0.2802 (4)	1.00116 (11)	0.06364 (14)	0.0218 (5)	
H5A	0.3806	1.0175	0.0388	0.026*	
H5B	0.2086	0.9750	0.0251	0.026*	
C6	0.1535 (4)	1.04789 (12)	0.08326 (16)	0.0263 (5)	
H6A	0.1014	1.0680	0.0346	0.039*	
H6B	0.2248	1.0741	0.1207	0.039*	
H6C	0.0530	1.0317	0.1071	0.039*	
C7	0.1289 (3)	0.66779 (11)	0.16308 (14)	0.0198 (5)	
C8	0.2339 (4)	0.60092 (11)	0.27098 (14)	0.0227 (5)	
H8A	0.3515	0.6003	0.2498	0.027*	
H8B	0.1977	0.5616	0.2792	0.027*	
C9	0.2634 (4)	0.63157 (12)	0.34969 (14)	0.0241 (5)	
H9A	0.2952	0.6714	0.3424	0.029*	
H9B	0.1500	0.6300	0.3739	0.029*	
C10	−0.1000 (4)	0.60685 (11)	0.21058 (15)	0.0232 (5)	
H10A	−0.1895	0.6373	0.1930	0.028*	
H10B	−0.1155	0.5956	0.2648	0.028*	
C11	−0.1435 (4)	0.55671 (12)	0.15499 (17)	0.0293 (6)	
H11A	−0.1319	0.5680	0.1004	0.035*	
H11B	−0.0530	0.5264	0.1717	0.035*	
C12	−0.3363 (4)	0.53524 (15)	0.15584 (18)	0.0370 (7)	
H12A	−0.3612	0.5032	0.1198	0.056*	
H12B	−0.4261	0.5651	0.1386	0.056*	
H12C	−0.3472	0.5235	0.2097	0.056*	
C13	−0.1430 (3)	0.78695 (11)	−0.09045 (15)	0.0223 (5)	
H13	−0.1766	0.8074	−0.0474	0.027*	
C14	−0.2656 (4)	0.78577 (12)	−0.16151 (16)	0.0267 (6)	
H14	−0.3805	0.8051	−0.1672	0.032*	
C15	−0.2165 (4)	0.75569 (11)	−0.22421 (16)	0.0267 (6)	
H15	−0.2978	0.7540	−0.2737	0.032*	
C16	−0.0475 (4)	0.72821 (11)	−0.21382 (14)	0.0230 (5)	
H16	−0.0107	0.7078	−0.2562	0.028*	
C17	0.0676 (3)	0.73098 (10)	−0.14026 (14)	0.0179 (5)	
C18	0.2499 (3)	0.70174 (10)	−0.12429 (14)	0.0193 (5)	
C19	0.5105 (4)	0.68052 (12)	−0.03162 (16)	0.0274 (6)	
H19	0.5775	0.6842	0.0207	0.033*	
C20	0.5869 (4)	0.64932 (13)	−0.08592 (18)	0.0342 (6)	
H20	0.7039	0.6316	−0.0714	0.041*	
C21	0.4884 (4)	0.64455 (14)	−0.16209 (18)	0.0370 (7)	
H21	0.5371	0.6231	−0.2006	0.044*	
C22	0.3192 (4)	0.67099 (12)	−0.18219 (16)	0.0286 (6)	
H22	0.2511	0.6683	−0.2345	0.034*	
C23	1.0929 (4)	0.93296 (12)	0.46333 (17)	0.0319 (6)	
C24	1.2471 (4)	0.93447 (15)	0.52810 (18)	0.0371 (7)	
H24A	1.2018	0.9298	0.5784	0.056*	
H24B	1.3111	0.9706	0.5280	0.056*	
H24C	1.3333	0.9040	0.5219	0.056*	

### Atomic displacement parameters (Å^2^)

**Table:**

	*U*^11^	*U*^22^	*U*^33^	*U*^12^	*U*^13^	*U*^23^
Cd	0.01970 (10)	0.01859 (11)	0.01415 (10)	−0.00001 (6)	0.00205 (7)	−0.00045 (6)
S1	0.0183 (3)	0.0203 (3)	0.0206 (3)	0.0005 (2)	0.0026 (2)	−0.0025 (2)
S2	0.0221 (3)	0.0212 (3)	0.0254 (3)	0.0018 (2)	0.0072 (2)	−0.0035 (2)
S3	0.0212 (3)	0.0273 (3)	0.0199 (3)	−0.0013 (2)	−0.0016 (2)	0.0036 (2)
S4	0.0203 (3)	0.0270 (3)	0.0190 (3)	−0.0008 (2)	0.0012 (2)	0.0055 (2)
O1	0.0331 (11)	0.0274 (10)	0.0200 (9)	−0.0029 (8)	−0.0002 (7)	−0.0002 (7)
O2	0.0453 (12)	0.0394 (12)	0.0185 (9)	0.0197 (10)	−0.0048 (8)	−0.0023 (8)
N1	0.0191 (10)	0.0201 (11)	0.0160 (9)	0.0003 (8)	0.0036 (7)	−0.0002 (7)
N2	0.0249 (11)	0.0221 (11)	0.0173 (10)	0.0020 (8)	0.0032 (8)	0.0022 (8)
N3	0.0193 (10)	0.0221 (11)	0.0201 (10)	−0.0003 (8)	0.0033 (8)	−0.0016 (8)
N4	0.0209 (10)	0.0146 (10)	0.0177 (10)	0.0002 (8)	0.0035 (8)	0.0016 (7)
N5	0.0482 (17)	0.0513 (18)	0.0362 (14)	−0.0215 (14)	−0.0089 (12)	0.0155 (12)
C1	0.0203 (12)	0.0222 (13)	0.0122 (10)	0.0008 (9)	0.0025 (8)	0.0015 (8)
C2	0.0206 (12)	0.0237 (13)	0.0200 (11)	−0.0041 (10)	0.0058 (9)	0.0008 (9)
C3	0.0217 (13)	0.0285 (14)	0.0244 (13)	−0.0020 (10)	0.0030 (10)	0.0030 (10)
C4	0.0222 (12)	0.0192 (12)	0.0174 (11)	−0.0006 (9)	0.0042 (9)	−0.0033 (9)
C5	0.0239 (12)	0.0223 (13)	0.0190 (11)	0.0004 (10)	0.0034 (9)	−0.0016 (9)
C6	0.0260 (13)	0.0255 (14)	0.0287 (13)	0.0059 (10)	0.0082 (10)	0.0010 (10)
C7	0.0228 (12)	0.0204 (13)	0.0161 (11)	0.0012 (9)	0.0036 (9)	−0.0015 (9)
C8	0.0297 (13)	0.0191 (13)	0.0191 (11)	0.0073 (10)	0.0041 (10)	0.0011 (9)
C9	0.0288 (13)	0.0252 (14)	0.0184 (11)	0.0071 (10)	0.0042 (10)	0.0003 (9)
C10	0.0273 (13)	0.0235 (13)	0.0202 (11)	−0.0002 (10)	0.0077 (9)	0.0021 (9)
C11	0.0292 (14)	0.0274 (15)	0.0317 (14)	−0.0008 (11)	0.0064 (11)	−0.0028 (11)
C12	0.0318 (16)	0.0453 (19)	0.0338 (15)	−0.0093 (13)	0.0051 (12)	−0.0038 (13)
C13	0.0211 (12)	0.0233 (14)	0.0234 (12)	0.0031 (10)	0.0064 (9)	0.0023 (9)
C14	0.0233 (13)	0.0287 (15)	0.0273 (13)	0.0044 (10)	0.0013 (10)	0.0061 (10)
C15	0.0285 (14)	0.0263 (14)	0.0227 (13)	−0.0028 (10)	−0.0032 (11)	0.0037 (10)
C16	0.0286 (14)	0.0233 (13)	0.0166 (11)	−0.0040 (10)	0.0021 (10)	−0.0001 (9)
C17	0.0184 (12)	0.0192 (12)	0.0167 (11)	−0.0038 (9)	0.0047 (9)	0.0006 (9)
C18	0.0214 (12)	0.0192 (12)	0.0180 (11)	−0.0002 (9)	0.0052 (9)	−0.0007 (9)
C19	0.0198 (12)	0.0322 (15)	0.0293 (13)	0.0055 (11)	0.0017 (10)	0.0006 (11)
C20	0.0232 (14)	0.0375 (17)	0.0433 (17)	0.0086 (12)	0.0099 (12)	0.0007 (13)
C21	0.0359 (16)	0.0408 (18)	0.0381 (16)	0.0100 (13)	0.0171 (13)	−0.0078 (13)
C22	0.0315 (14)	0.0320 (16)	0.0238 (13)	0.0009 (11)	0.0087 (11)	−0.0069 (11)
C23	0.0374 (16)	0.0254 (15)	0.0330 (15)	−0.0055 (12)	0.0062 (12)	0.0069 (11)
C24	0.0341 (16)	0.0395 (18)	0.0361 (16)	0.0007 (13)	0.0013 (12)	0.0090 (13)

### Geometric parameters (Å, °)

**Table:**

Cd—N3	2.361 (2)	C6—H6B	0.9800
Cd—N4	2.406 (2)	C6—H6C	0.9800
Cd—S1	2.5872 (7)	C8—C9	1.515 (3)
Cd—S3	2.6539 (7)	C8—H8A	0.9900
Cd—S4	2.6704 (7)	C8—H8B	0.9900
Cd—S2	2.7816 (7)	C9—H9A	0.9900
S1—C1	1.736 (2)	C9—H9B	0.9900
S2—C1	1.723 (2)	C10—C11	1.527 (4)
S3—C7	1.723 (3)	C10—H10A	0.9900
S4—C7	1.724 (2)	C10—H10B	0.9900
O1—C3	1.426 (3)	C11—C12	1.505 (4)
O1—H1O	0.8400	C11—H11A	0.9900
O2—C9	1.427 (3)	C11—H11B	0.9900
O2—H2O	0.8400	C12—H12A	0.9800
N1—C1	1.340 (3)	C12—H12B	0.9800
N1—C2	1.472 (3)	C12—H12C	0.9800
N1—C4	1.475 (3)	C13—C14	1.384 (4)
N2—C7	1.337 (3)	C13—H13	0.9500
N2—C10	1.467 (3)	C14—C15	1.388 (4)
N2—C8	1.478 (3)	C14—H14	0.9500
N3—C19	1.345 (3)	C15—C16	1.385 (4)
N3—C18	1.345 (3)	C15—H15	0.9500
N4—C13	1.340 (3)	C16—C17	1.393 (3)
N4—C17	1.346 (3)	C16—H16	0.9500
N5—C23	1.141 (4)	C17—C18	1.490 (3)
C2—C3	1.515 (3)	C18—C22	1.395 (3)
C2—H2A	0.9900	C19—C20	1.381 (4)
C2—H2B	0.9900	C19—H19	0.9500
C3—H3A	0.9900	C20—C21	1.383 (4)
C3—H3B	0.9900	C20—H20	0.9500
C4—C5	1.526 (3)	C21—C22	1.381 (4)
C4—H4A	0.9900	C21—H21	0.9500
C4—H4B	0.9900	C22—H22	0.9500
C5—C6	1.523 (3)	C23—C24	1.443 (4)
C5—H5A	0.9900	C24—H24A	0.9800
C5—H5B	0.9900	C24—H24B	0.9800
C6—H6A	0.9800	C24—H24C	0.9800
			
N3—Cd—N4	68.37 (7)	S3—C7—S4	119.26 (14)
N3—Cd—S1	141.84 (5)	N2—C8—C9	111.5 (2)
N4—Cd—S1	98.70 (5)	N2—C8—H8A	109.3
N3—Cd—S3	96.53 (5)	C9—C8—H8A	109.3
N4—Cd—S3	143.78 (5)	N2—C8—H8B	109.3
S1—Cd—S3	111.54 (2)	C9—C8—H8B	109.3
N3—Cd—S4	106.87 (5)	H8A—C8—H8B	108.0
N4—Cd—S4	84.70 (5)	O2—C9—C8	105.9 (2)
S1—Cd—S4	107.40 (2)	O2—C9—H9A	110.5
S3—Cd—S4	67.92 (2)	C8—C9—H9A	110.6
N3—Cd—S2	86.66 (5)	O2—C9—H9B	110.5
N4—Cd—S2	118.12 (5)	C8—C9—H9B	110.5
S1—Cd—S2	67.51 (2)	H9A—C9—H9B	108.7
S3—Cd—S2	92.38 (2)	N2—C10—C11	112.4 (2)
S4—Cd—S2	156.855 (19)	N2—C10—H10A	109.1
C1—S1—Cd	89.45 (8)	C11—C10—H10A	109.1
C1—S2—Cd	83.49 (9)	N2—C10—H10B	109.1
C7—S3—Cd	86.69 (8)	C11—C10—H10B	109.1
C7—S4—Cd	86.13 (9)	H10A—C10—H10B	107.9
C3—O1—H1O	105.4	C12—C11—C10	110.9 (2)
C9—O2—H2O	111.9	C12—C11—H11A	109.5
C1—N1—C2	122.3 (2)	C10—C11—H11A	109.5
C1—N1—C4	121.6 (2)	C12—C11—H11B	109.5
C2—N1—C4	115.9 (2)	C10—C11—H11B	109.5
C7—N2—C10	122.5 (2)	H11A—C11—H11B	108.0
C7—N2—C8	121.8 (2)	C11—C12—H12A	109.5
C10—N2—C8	115.6 (2)	C11—C12—H12B	109.5
C19—N3—C18	119.2 (2)	H12A—C12—H12B	109.5
C19—N3—Cd	120.38 (17)	C11—C12—H12C	109.5
C18—N3—Cd	120.40 (16)	H12A—C12—H12C	109.5
C13—N4—C17	118.6 (2)	H12B—C12—H12C	109.5
C13—N4—Cd	122.48 (16)	N4—C13—C14	123.0 (2)
C17—N4—Cd	118.74 (16)	N4—C13—H13	118.5
N1—C1—S2	120.62 (18)	C14—C13—H13	118.5
N1—C1—S1	119.92 (18)	C13—C14—C15	118.4 (2)
S2—C1—S1	119.46 (14)	C13—C14—H14	120.8
N1—C2—C3	112.5 (2)	C15—C14—H14	120.8
N1—C2—H2A	109.1	C16—C15—C14	119.2 (2)
C3—C2—H2A	109.1	C16—C15—H15	120.4
N1—C2—H2B	109.1	C14—C15—H15	120.4
C3—C2—H2B	109.1	C15—C16—C17	119.0 (2)
H2A—C2—H2B	107.8	C15—C16—H16	120.5
O1—C3—C2	108.4 (2)	C17—C16—H16	120.5
O1—C3—H3A	110.0	N4—C17—C16	121.8 (2)
C2—C3—H3A	110.0	N4—C17—C18	116.2 (2)
O1—C3—H3B	110.0	C16—C17—C18	122.0 (2)
C2—C3—H3B	110.0	N3—C18—C22	121.2 (2)
H3A—C3—H3B	108.4	N3—C18—C17	116.2 (2)
N1—C4—C5	111.54 (19)	C22—C18—C17	122.6 (2)
N1—C4—H4A	109.3	N3—C19—C20	122.6 (3)
C5—C4—H4A	109.3	N3—C19—H19	118.7
N1—C4—H4B	109.3	C20—C19—H19	118.7
C5—C4—H4B	109.3	C19—C20—C21	118.2 (3)
H4A—C4—H4B	108.0	C19—C20—H20	120.9
C6—C5—C4	111.1 (2)	C21—C20—H20	120.9
C6—C5—H5A	109.4	C22—C21—C20	120.0 (3)
C4—C5—H5A	109.4	C22—C21—H21	120.0
C6—C5—H5B	109.4	C20—C21—H21	120.0
C4—C5—H5B	109.4	C21—C22—C18	118.8 (3)
H5A—C5—H5B	108.0	C21—C22—H22	120.6
C5—C6—H6A	109.5	C18—C22—H22	120.6
C5—C6—H6B	109.5	N5—C23—C24	179.1 (3)
H6A—C6—H6B	109.5	C23—C24—H24A	109.5
C5—C6—H6C	109.5	C23—C24—H24B	109.5
H6A—C6—H6C	109.5	H24A—C24—H24B	109.5
H6B—C6—H6C	109.5	C23—C24—H24C	109.5
N2—C7—S3	120.65 (19)	H24A—C24—H24C	109.5
N2—C7—S4	120.09 (19)	H24B—C24—H24C	109.5
			
N3—Cd—S1—C1	−52.88 (11)	Cd—S1—C1—S2	3.16 (13)
N4—Cd—S1—C1	−118.76 (9)	C1—N1—C2—C3	−90.2 (3)
S3—Cd—S1—C1	81.54 (8)	C4—N1—C2—C3	95.2 (3)
S4—Cd—S1—C1	154.10 (8)	N1—C2—C3—O1	−63.7 (3)
S2—Cd—S1—C1	−1.85 (7)	C1—N1—C4—C5	−83.9 (3)
N3—Cd—S2—C1	153.10 (9)	C2—N1—C4—C5	90.8 (2)
N4—Cd—S2—C1	89.84 (9)	N1—C4—C5—C6	177.9 (2)
S1—Cd—S2—C1	1.87 (8)	C10—N2—C7—S3	179.42 (18)
S3—Cd—S2—C1	−110.49 (8)	C8—N2—C7—S3	−3.5 (3)
S4—Cd—S2—C1	−79.80 (9)	C10—N2—C7—S4	−1.5 (3)
N3—Cd—S3—C7	−105.50 (10)	C8—N2—C7—S4	175.58 (18)
N4—Cd—S3—C7	−43.64 (12)	Cd—S3—C7—N2	178.9 (2)
S1—Cd—S3—C7	100.87 (8)	Cd—S3—C7—S4	−0.15 (14)
S4—Cd—S3—C7	0.09 (8)	Cd—S4—C7—N2	−178.9 (2)
S2—Cd—S3—C7	167.59 (8)	Cd—S4—C7—S3	0.15 (13)
N3—Cd—S4—C7	90.20 (10)	C7—N2—C8—C9	91.3 (3)
N4—Cd—S4—C7	155.69 (10)	C10—N2—C8—C9	−91.5 (3)
S1—Cd—S4—C7	−106.83 (8)	N2—C8—C9—O2	−176.7 (2)
S3—Cd—S4—C7	−0.09 (8)	C7—N2—C10—C11	89.8 (3)
S2—Cd—S4—C7	−33.49 (10)	C8—N2—C10—C11	−87.4 (3)
N4—Cd—N3—C19	178.4 (2)	N2—C10—C11—C12	178.6 (2)
S1—Cd—N3—C19	102.4 (2)	C17—N4—C13—C14	−0.8 (4)
S3—Cd—N3—C19	−35.7 (2)	Cd—N4—C13—C14	−175.62 (19)
S4—Cd—N3—C19	−104.5 (2)	N4—C13—C14—C15	0.2 (4)
S2—Cd—N3—C19	56.3 (2)	C13—C14—C15—C16	−0.2 (4)
N4—Cd—N3—C18	−1.17 (17)	C14—C15—C16—C17	0.8 (4)
S1—Cd—N3—C18	−77.2 (2)	C13—N4—C17—C16	1.4 (4)
S3—Cd—N3—C18	144.74 (18)	Cd—N4—C17—C16	176.40 (18)
S4—Cd—N3—C18	75.88 (18)	C13—N4—C17—C18	−179.0 (2)
S2—Cd—N3—C18	−123.25 (18)	Cd—N4—C17—C18	−4.0 (3)
N3—Cd—N4—C13	177.6 (2)	C15—C16—C17—N4	−1.4 (4)
S1—Cd—N4—C13	−39.79 (19)	C15—C16—C17—C18	179.0 (2)
S3—Cd—N4—C13	107.09 (19)	C19—N3—C18—C22	0.9 (4)
S4—Cd—N4—C13	67.05 (19)	Cd—N3—C18—C22	−179.6 (2)
S2—Cd—N4—C13	−108.88 (19)	C19—N3—C18—C17	−179.9 (2)
N3—Cd—N4—C17	2.78 (17)	Cd—N3—C18—C17	−0.3 (3)
S1—Cd—N4—C17	145.43 (16)	N4—C17—C18—N3	2.9 (3)
S3—Cd—N4—C17	−67.7 (2)	C16—C17—C18—N3	−177.5 (2)
S4—Cd—N4—C17	−107.73 (17)	N4—C17—C18—C22	−177.9 (2)
S2—Cd—N4—C17	76.34 (18)	C16—C17—C18—C22	1.7 (4)
C2—N1—C1—S2	0.1 (3)	C18—N3—C19—C20	−1.0 (4)
C4—N1—C1—S2	174.40 (16)	Cd—N3—C19—C20	179.4 (2)
C2—N1—C1—S1	−179.15 (17)	N3—C19—C20—C21	0.4 (5)
C4—N1—C1—S1	−4.8 (3)	C19—C20—C21—C22	0.5 (5)
Cd—S2—C1—N1	177.82 (19)	C20—C21—C22—C18	−0.7 (5)
Cd—S2—C1—S1	−2.96 (12)	N3—C18—C22—C21	0.0 (4)
Cd—S1—C1—N1	−177.61 (18)	C17—C18—C22—C21	−179.2 (3)

### Hydrogen-bond geometry (Å, °)

**Table:**

*D*—H···*A*	*D*—H	H···*A*	*D*···*A*	*D*—H···*A*
O1—H1o···N5	0.84	2.06	2.898 (3)	174
O2—H2o···S2^i^	0.84	2.55	3.388 (2)	175

Symmetry codes: (i) *x*, −*y*+3/2, *z*+1/2.

## References

[bb1] Benson, R. E., Ellis, C. A., Lewis, C. E. & Tiekink, E. R. T. (2007). *CrystEngComm*, **9**, 930–940.

[bb2] Beurskens, P. T., Admiraal, G., Beurskens, G., Bosman, W. P., Garcia-Granda, S., Gould, R. O., Smits, J. M. M. & Smykalla, C. (1992). *The *DIRDIF* Program System*. Technical Report. Crystallography Laboratory, University of Nijmegen, The Netherlands.

[bb3] Brandenburg, K. (2006). *DIAMOND*. Crystal Impact GbR, Bonn, Germany.

[bb4] Chen, D., Lai, C. S. & Tiekink, E. R. T. (2006). *CrystEngComm*, **8**, 51–58.

[bb5] Higashi, T. (1995). *ABSCOR*. Rigaku Corporation, Tokyo, Japan.

[bb6] Johnson, C. K. (1976). *ORTEPII*. Report ORNL-5138. Oak Ridge National Laboratory, Tennessee, USA.

[bb7] Lai, C. S., Lim, Y. X., Yap, T. C. & Tiekink, E. R. T. (2002). *CrystEngComm*, **4**, 596–600.

[bb8] Lai, C. S. & Tiekink, E. R. T. (2004). *CrystEngComm*, **6**, 593–605.

[bb9] Rigaku/MSC (2005). *CrystalClear*. Rigaku/MSC Inc., The Woodlands, Texas, USA.

[bb10] Sheldrick, G. M. (2008). *Acta Cryst.* A**64**, 112–122. 10.1107/S0108767307043930 18156677

[bb11] Tiekink, E. R. T. (2003). *CrystEngComm*, **5**, 101–113.

[bb12] Westrip, S. P. (2009). *publCIF*. In preparation.

